# Recent Advances on Nutrition in Treatment of Acute Pancreatitis

**DOI:** 10.3389/fimmu.2017.00762

**Published:** 2017-06-30

**Authors:** Li-Long Pan, Jiahong Li, Muhammad Shamoon, Madhav Bhatia, Jia Sun

**Affiliations:** ^1^School of Medicine, Jiangnan University, Wuxi, China; ^2^State Key Laboratory of Food Science and Technology, Jiangnan University, Wuxi, China; ^3^Nutrition and Immunology Laboratory, School of Food Science and Technology, Jiangnan University, Wuxi, China; ^4^Inflammation Research Group, Department of Pathology, University of Otago, Christchurch, New Zealand

**Keywords:** clinical management of acute pancreatitis, nutritional interventions, probiotics, prebiotics, vitamins, amino acids, omega-3 fatty acids

## Abstract

Acute pancreatitis (AP) is a common abdominal acute inflammatory disorder and the leading cause of hospital admission for gastrointestinal disorders in many countries. Clinical manifestations of AP vary from self-limiting local inflammation to devastating systemic pathological conditions causing significant morbidity and mortality. To date, despite extensive efforts in translating promising experimental therapeutic targets in clinical trials, disease-specific effective remedy remains obscure, and supportive care has still been the primary treatment for this disease. Emerging evidence, in light of the current state of pathophysiology of AP, has highlighted that strategic initiation of nutrition with appropriate nutrient supplementation are key to limit local inflammation and to prevent or manage AP-associated complications. The current review focuses on recent advances on nutritional interventions including enteral versus parenteral nutrition strategies, and nutritional supplements such as probiotics, glutamine, omega-3 fatty acids, and vitamins in clinical AP, hoping to advance current knowledge and practice related to nutrition and nutritional supplements in clinical management of AP.

## Introduction

Acute pancreatitis is the leading cause of acute hospital admission for gastrointestinal disorders in many countries, and its incidence continues to raise worldwide (1–3). The annual incidence of AP ranges from 13 to 45 cases per 100,000 population with the global estimate of 33.74 cases per 100,000 population, causing uneven burden across the globe. The health-care cost in the United States is reported to be $2.5 billion (1, 4, 5). Gallstones and alcoholism are the long-established two most common etiological factors, and other risk factors such as genetic predisposition, drugs, smoking, type 2 diabetes, and endoscopic retrograde cholangiopancreatography play a part (1, 3, 6). Clinical manifestations of AP vary from a mild edematous form to severe fulminant pancreatitis with potential devastating complications (7). Severity of AP is stratified into three categories: mild, moderately severe, and severe (Table 1). The overall mortality ranges from 5 to 20% depending on severity (8, 9). In patients who develop severe necrotizing pancreatitis, mortality is approximately 15%. In cases of infection of pancreatic necrosis and multi-organ failure, mortality can be as high as 30% (8). In China, the overall mortality rate of severe AP patients was estimated to be 11.8% (7). Up to date, a major challenge in search of targeted pharmacological therapy specific to AP, despite extensive efforts, is due to heterogeneous etiological factors and varying clinical manifestations associated with this condition (9, 10).

**Table 1:** AP classification.

Classification	Severity	Local complications	Systemic complications			Reference
			TOF	POF	EPC	
Atlanta 2012^a^	Mild	×	×	×	×	(10, 11)
	Moderate	√	√	×	√	
	Severe	√	×	√	√/×	

Determinant based^b^	Mild	×	×	×	N/A	
	Moderate	Sterile	√	×	N/A	
	Severe	Infected	√	√	N/A	
	Critical	Infected	×	√	N/A

*AP, acute pancreatitis; EPC, exacerbation of preexisting comorbidity; N/A, not applicable; POF, persistent organ failure; TOF, transient organ failure; √, yes; ×, no*.

*^a^In Atlanta 2012, local complications are subcategorized (interstitial edematous, necrotizing pancreatitis, infected necrotizing pancreatitis, other local complications, etc.), whereas systemic complications are defined as TOF or POF or an EPC (organ failure persisting for >48 h; three organ systems = renal, respiratory, cardiovascular; Marshal score ≥2)*.

*^b^Sepsis-related organ failure assessment scoring system is used to define organ failure, and for severe pancreatitis, either POF or infected necrosis is mandatory*.

**Figure 1:**
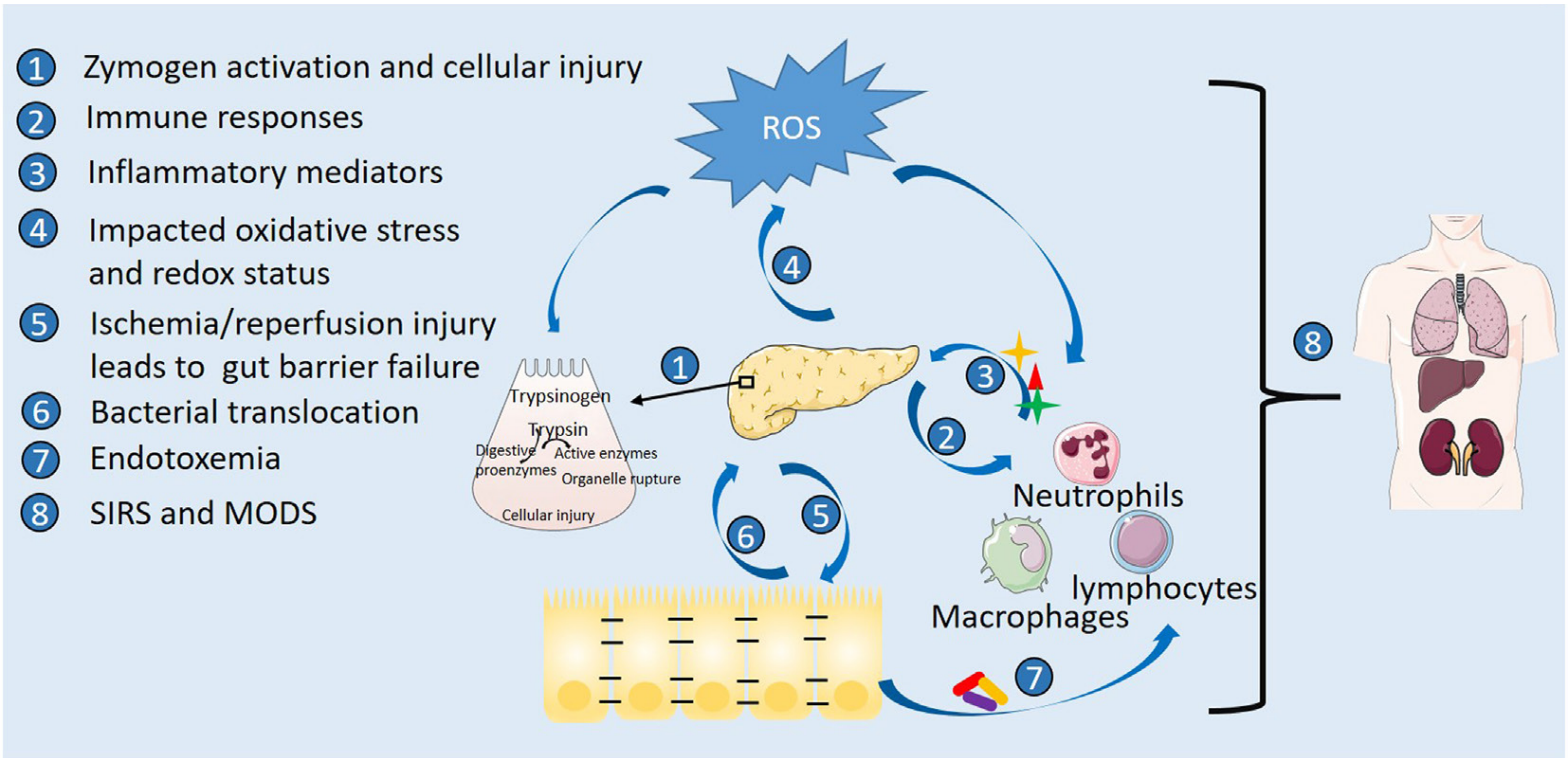
Pathophysiology of acute pancreatitis highlighting sites of action by nutrition. Etiological stress triggers premature activation of digestive zymogens and intra-acinar cellular injury with accompanying oxidative stress. Involvement of immune cells with released inflammatory mediators and amplified oxidative stress exacerbate the inflammatory cascade. Gut inflammation and barrier failure occur following systemic inflammatory responses, vascular disturbance, and ischemia/reperfusion injury secondary to pancreatic inflammation. Disrupted barrier function further leads to bacterial translocation, pancreatic infection and necrosis, and endotoxemia, ultimately responsible for multiple organ dysfunction syndrome (MODS) and death.

Pathophysiology of AP encompasses complex cascaded events of acinar cell inflammation, involvement of immune system, and systemic pathological outcomes (12) (Figure 1). Premature activation of intra-acinar digestive zymogens is one of the early hallmarks of AP. The resultant autodigestion of pancreas leads to release of pro-inflammatory mediators such as tumor necrosis factor-α, interleukin (IL)-1β, IL-6, which intermingle with microcirculation, causing increased vascular permeability, edema, hemorrhage, and necrosis of pancreas (13–15). Profound acinar cell injury and amplified inflammatory responses give rise to systemic inflammatory response syndrome (SIRS) and multiple organ dysfunction syndrome (MODS), ultimately responsible for AP-associated mortality (16–18). The immune system is thought to play an important role in the disease pathogenesis of AP. Complex immunological events underlie progression of AP (12, 19). Dysregulated immune responses during AP include increased leukocyte counts, migration and activation of pro-inflammatory innate immune cells (neutrophils and macrophages) as well as depletion of T-lymphocytes and raised levels of plasma pro-inflammatory cytokines (12). Innate immune cells and derived inflammatory mediators as potential therapeutic targets have thus drawn much attention.

Better understanding of the pathophysiology of AP has drawn research efforts to reestablish the immune and organ/tissue homeostasis in clinical AP and toward the development of new intervention strategies (20). With still obscure disease-specific pharmacological therapies, developing managing strategies from randomized clinical trials are critical in the prevention of systemic complications during severe AP. Nutrition support and intervention is an important part of clinical management of patients with AP (21, 22).

## Nutritional Intervention in Clinical AP

**Figure 2:**
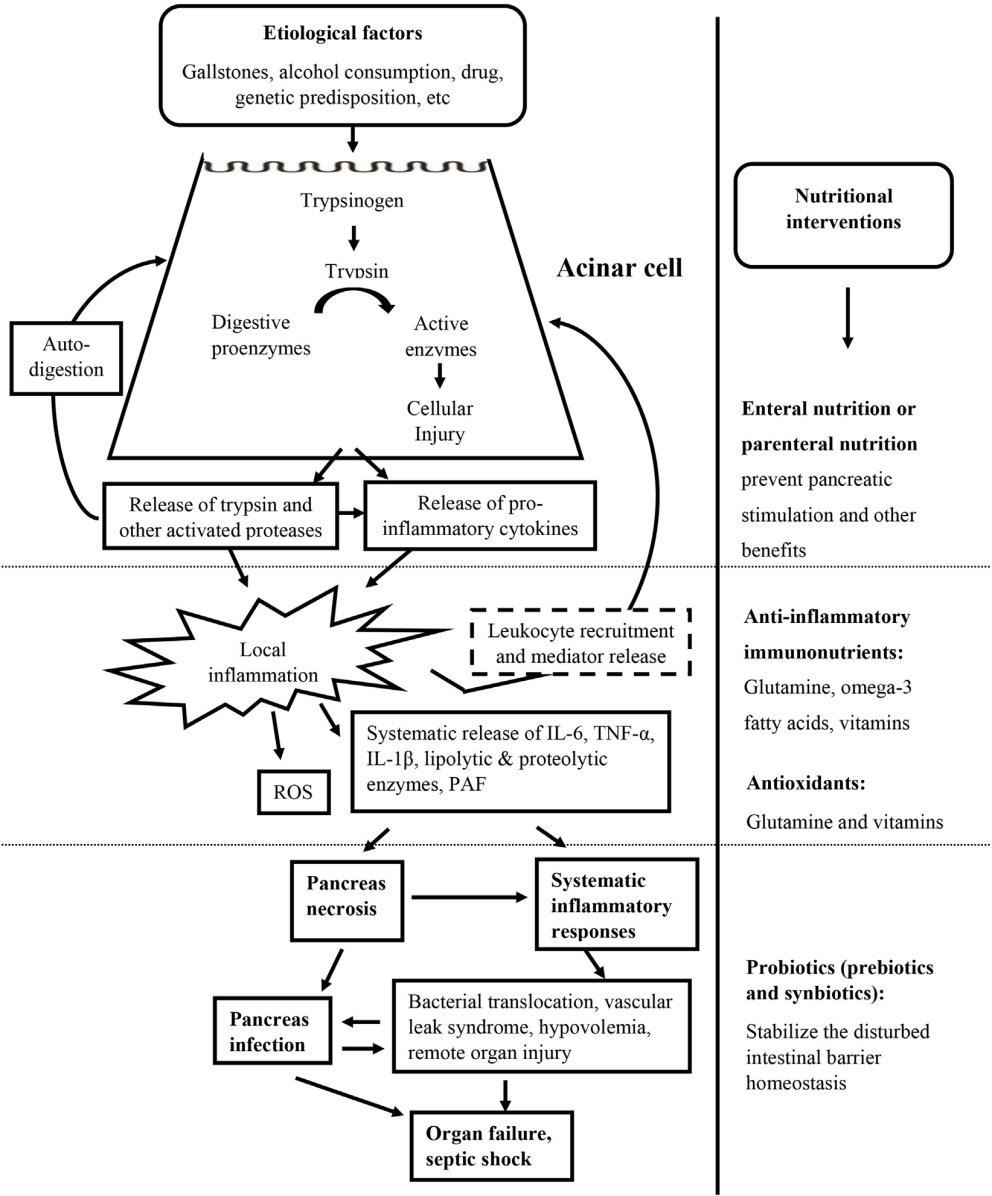
Targeted nutritional interventions during the whole episode of acute pancreatitis. Targeted nutritional interventions: enteral or parental nutrition and nutritional supplements including anti-inflammatory immunonutrients, antioxidants, and probiotics are presented at the administration stage.

Nutrition and nutritional supplements have demonstrated necessity and importance not only in restoring energy balance but also in maintaining gut barrier function and providing important immunomodulatory and antioxidant effects (Figure 2). The gut is an important secondary organ and also a site of starting severe systemic complications during AP. Intestinal barrier dysfunction is associated with translocation of bacteria and their inflammatory and toxic products, responsible for infection of the necrotic pancreas and systemic inflammatory responses. Therefore, maintaining the integrity of the gut barrier in the small intestine is one of the main goals in early-phase treatment of severe AP (23). Optimal nutritional support in AP has been under debate for decades. Bowl at rest (*nothing by mouth*) strategy has been implemented conventionally to treat AP (24, 25). However, dietary restrictions exacerbate patient’s malnutrition due to imbalance between reduced food intake and higher nutritional requirements, leading to further catabolism, bacterial translocation (26), and ultimate mortality (27). Evidence of clinical trials has demonstrated parenteral nutrition (PN) in preventing pancreatic stimulation and many benefits of enteral nutrition (EN). However, in daily practice, it remains challenging to predict whether EN will be tolerated in patients with AP (8).

Strategic approaches to include nutritional supplements have also been attempted to provide additional immune regulatory and antioxidative effects. Probiotics and prebiotics have been shown to stabilize the disturbed intestinal barrier homeostasis and be beneficial in reducing the infection rate in primary clinical trials (28–31). Due to the immunosuppressive and inflammatory nature of the disease, immunonutrients like glutamine and omega-3 fatty acids (ω-3 FAs) have been added to parenteral or enteral formulas to modulate immune functions, suppress the hyper inflammatory responses, and reestablish tissue and organ homeostasis in clinical practice (21, 32, 33). Supplements with antioxidative properties like glutamine and vitamin C have also been suggested to provide additional beneficial effects (34).

The review aims to provide a comprehensive chronological review on latest clinical trials on EN versus PN strategies and nutritional supplements including probiotics (prebiotics and synbiotics), glutamine, ω-3 FAs, and vitamins, hoping to provide the basis for future development of nutritional strategies in clinical AP.

## EN versus PN

Traditionally, AP patients were maintained on *nil per os* or *nothing per mouth* treatment until resolution of pain or normalization of pancreatic enzymes to allow the pancreas to rest (35). Currently, it is widely accepted that early EN may be critical to improve AP-associated malnutrition and the overall outcomes, as bowel rest is associated with intestinal mucosal atrophy and increased infectious complications (9). Gut barrier dysfunction is found in approximately 60% of patients with AP (8, 36). Importantly, EN exerts immunomodulatory effects to preserve gut mucosa integrity, stimulate intestinal motility, and reduce bacterial overgrowth (8, 37). A randomized clinical study demonstrated that immediate oral feeding in patients with mild AP was feasible and safe and accelerated recovery without adverse gastrointestinal events (38). Another randomized controlled trial supported early-stage introduction of initial oral nutrition with either a clear liquid diet or a low-fat solid diet for patients who developed mild AP (39). In these patients, if oral intake is not tolerated, enteral feeding is recommended (9). In patients with severe AP or predicted severe AP, EN with oral or tube feeding thought to preserve the gut barrier function to prevent bacterial translocation is preferred over PN. A multicenter randomized study in the *New England Journal of Medicine* demonstrated that early tube feeding and oral diet after 72 h are equivalent in reducing infection rates or death in AP patients at high risk for complications (40). A Cochrane meta-analysis of eight randomized controlled studies found that EN reduced mortality, systemic infections, and multiorgan failure among patients with AP as compared to PN (41). Another meta-analysis of 381 patients confirmed the benefit of EN versus PN support in patients with severe AP with lower mortality, fewer infectious complications, decreased organ failure and surgical intervention rate (42). Over the optimal route of EN, several trials have suggested the nasogastric route as an alternative to nasoduodenal or nasojejunal routes (43). Multiple randomized controlled trials involving 157 patients with predicted severe AP demonstrated that nasogastric feeding was safe and well tolerated compared with nasojejunal feeding (44). Given its demonstrated beneficial outcomes, it remains challenging to predict whether EN will be tolerated in patients with AP (8). However, as shown by multiple randomized trials that have associated total PN (TPN) with risks of infection and other complications (35), PN should still be minimized unless the enteral route is not available, not tolerated, or not meeting caloric requirements.

## Nutritional Supplements

### Probiotics, Prebiotics, and Synbiotics

Changes in intestinal motility and microbiome, immune response, and mucosal barrier function during AP lead to bacterial translocation and subsequent pancreatic necrosis infection, which is one of the principal causes of complications and death in severe AP patients (45). Potential roles of probiotics have been proposed for immunomodulatory and health-promoting benefits to restore the gut integrity, modulate immune responses against invading pathogens, and prevent proliferation of harmful bacteria beyond those of basic nutrition, which have been evaluated in a number of clinical trials (Table 2).

**Table 2:** Characteristics of clinical trials on probiotic treatment in AP.

Reference	Probiotic(s) or prebiotic(s) tested	Comparison groups	Gut barrier permeability		Systemic complications				
			Methods	Results	Infected necrosis	SIRS	MODS	Infection	Mortality
Olah et al. (46)	*Lactobacillus plantarum* 299 plus oat fiber (10^9^ × 2/daily dose)	EN + symbiotic + fibers versus EN + heat-inactivated symbiotic + fibers	–	–	No difference	No difference	No difference	↓ pancreatic infection requiring operation in the probiotic arm	No difference

Kecskes et al. (47)	*L. plantarum* 299 plus oat fiber	EN + symbiotic + fibers versus EN + heat-inactivated symbiotic + fibers	–	–	↓ in symbiotic arm	–		–	–

Olah et al. (48)	Multistrain (40 × 10^9^/daily dose) and multifibers	EN + fibers versus EN + fibers + symbiotic	–	–	↓ in symbiotic arm	↓ SIRS + MODS in symbiotic arm		↓ surgical interventions in the probiotic arm	No difference

Qin et al. (49)	*L. plantarum* (unspecified strain) (10^10^/daily dose)	TPN versus partial PN + EN + probiotics	Lactulose/rhamnose urinary excretion	↓ in the probiotic arm	–	↓ SIRS in the probiotic arm	↓ MODS in the probiotic arm	↓ infective complications in the probiotic arm	No difference

Karakan et al. (50)	Multifibers	EN + multifibers versus EN	–	–	–	No difference	No difference	–	No difference

Besselink et al. (51)	Multistrain product (10^10^/daily dose) plus maltodextrins and cornstarch	EN + placebo versus EN + probiotics	–	–	No difference	–	↑ MODS in the probiotic arm	No difference	↑ in the probiotic arm due to NOMI

Besselink et al. (52)	Multistrain product (10^10^/daily dose)	EN + placebo versus EN + probiotics	PEG urinary excretion	No difference	–	–	–	–	–

Sharma et al. (53)	Multistrain product (10^10^/daily dose)	Placebo versus probiotics (through the current mode of feeding)	Lactulose/rhamnose urinary excretion	No difference	–	–	No difference	↓ endotoxin core antibody IgG, IgM in the probiotic arm	No difference

Cui et al. (54)	Multistrain product 1 × 10^11^/12 h	PN versus EN versus EN + probiotics (PN)	–	–	↓ in the EN arm and EN + probiotics arm	–	–	–	No difference

*AP, acute pancreatitis; EN, enteral nutrition; MODS, multiple organ dysfunction syndrome; PN, parenteral nutrition; SIRS, systemic inflammatory response syndrome; TPN, total parenteral nutrition*.

An early indication of beneficial effects of synbiotics on severe AP-associated endotoxemia came from a randomized, double-blind clinical trial with 45 patients receiving either live or heat-inactivated *Lactobacillus plantarum 299* with oat fiber supplement as early EN. The results suggested that supplementary combined pre- and probiotics was effective in reducing infected pancreatic necrosis and surgical interventions (46, 47). The findings were subsequently supported and extended by a larger study with 62 patients on the Synbiotic 2000 formulated early EN with four different types of prebiotics (inulin, beta-glucan, resistant starch, and pectin) and probiotics (four different *Lactobacilli* preparations). Patients receiving synbiotic therapy had reduced total incidence of SIRS and lower rates of organ failure, supporting that early EN with synbiotics may prevent organ dysfunctions in the late phase of severe AP (48). The effects of *L. plantarum* only enteral feeding were evaluated in 76 patients with AP. Overall, the patients with ecoimmunonutrition showed attenuated disease severity, improved intestinal permeability, and better clinical outcomes (49). Prebiotic fiber alone supplementation with EN assessed in a randomized, double-blind study with 30 consecutive severe AP patients was found to shorten hospital stay, duration of nutrition therapy, and reduce the acute phase response and overall complications compared to standard EN therapy (50). Probiotic prophylaxis in severe AP has been contraindicated. The Dutch Acute Pancreatitis Study Group reported in PROPATRIA, a multicenter, randomized, double-blind, placebo-controlled trial with in a total of 200 patients with predicted severe AP that multispecies probiotic (Ecologic 641: six probiotic strains) prophylaxis did not reduce the risk of infectious complications and was associated with an increased risk of mortality (55, 56), although overall this combination of probiotic strains reduced bacterial translocation (52). Following studies involving multispecies probiotic supplementation with EN early abandoned after the publication of PROPATRIA study seemed to support the results that no significant trend was identified for an effect of probiotics on gut permeability or endotoxemia in AP (53, 57), although a positive effect was observed with reduced endotoxin levels (57). Recently, a local study of 70 patients with severe AP comparing PN, EN and EN with addition of the probiotic *Bifidobacterium* found that early EN with *Bifidobacterium* resulted in lower levels of pro-inflammatory cytokines, improved gastrointestinal function, reduced complications, and shorter hospital stay in patients with severe AP (54). These data suggest the potential of single specific probiotic strains supplemented, which however should be further evaluated by validated clinical trials before their beneficial effects could be confirmed.

### Glutamine

Glutamine is an important constituent of intra and extracellular amino acid pool, with immune modulatory and antioxidant effects, and its depletion has been demonstrated in critical illness (58). Glutamine improves immune cell functions and contributes to antioxidative defenses. It can also support the intestinal integrity and decrease bacterial translocation; hence reduce systemic inflammatory responses and sepsis, which are important in critical illnesses such as AP (33).

An early randomized, controlled study with 28 AP patients received either a standard TPN or an isonitrogen, isocaloric TPN containing 0.3 g/kg l-alanine-l-glutamine demonstrated that glutamine supplementation with TPN was associated with a significant increase of cholinesterase, albumin, and lymphocyte count in AP as well a decrease of C-reactive protein compared to standard TPN. AP patients receiving glutamine was associated with a reduced length of TPN and a trend of reduced length of hospital stay, suggesting that glutamine substitution in TPN is beneficial in patients with AP (59). The effects of glutamine enriched (0.3 g/kg/day) TPN when further evaluated in 40 patients with AP. Beneficial effects of glutamine supplementation to TPN were found on acute pancreatic responses with serum lipase, amylase activities, and C-reactive protein levels decreased and the prevention of complications in patients with AP (59). Later, the effect of parenteral glutamine on recovery from severe AP was more thoroughly investigated in a randomized trial with 44 patients. l-alanyl-l-glutamine-supplemented PN increased serum IL-10 levels, improved nitrogen balance, and decreased infectious morbidity in patients with severe AP (60). Enterally, supplementation of glutamine and arginine in patients diagnosed of AP and predicted to develop a severe course was found to improve gut barrier function by reducing the gut permeability and decreasing plasma endotoxin level in the early stage of severe AP (61). Other than glutamine supplemented with TPN and EN, intravenously administered glutamine with early nasojejunal nutrition was also evaluated. In a randomized study, 45 patients with severe AP received glutamine or normal amino acid solution together with nasojejunal nutrition. The results demonstrated that the glutamine-receiving group showed signs of improvement in all end-point measurements including the rate of pancreas-specific infectious complications, organ failure, length of hospital stay, and mortality rate; and statistical significant difference was noted only in the length of hospital stay (62). Furthermore, a randomized trial compared early versus late intravenous infusion of alanylglutamine dipeptide in 76 patients with severe AP and demonstrated that early-stage intervention achieved a better clinical outcome: shortened duration of hospitalization, reduced rate of infection, organ dysfunction, need for surgery, and mortality, compared to the late treatment (63). More recently, glutamine supplemented in combination with normal saline and hydroxyethyl starch in resuscitation fluids were more efficient in relieving inflammation and sustaining the intestinal barrier in patients with severe AP (64). Two recent meta-analysis studies of randomized controlled trials demonstrated that glutamine supplementation resulted in significantly reduced mortality and complications (65, 66). Further analysis suggested a clear advantage for glutamine supplementation in patients who received TPN. In contrast, patients with AP who received EN did not require glutamine supplementation (65). Finally, oral glutamine supplementation did not seem to confer any significant effect on gut permeability and endotoxemia in severe AP (67). Characteristics of clinical studies on glutamine supplementation included in this review have been summarized in Table 3. Together, while glutamine supplementation with TPN shows promising clinical outcomes, enteral glutamine supplementation needs to be investigated in future.

**Table 3:** Characteristics of clinical trials on glutamine as the nutritional supplement in AP.

Reference	Subjects/regions	Dosage (g/kg BW/day)	Method of assessment	AD-EN or PN interval (h)	Duration of EN or PN (days)		Infectious complication (*n*/*N*)		Mortality (*n*/*N*)		DOS (median or days mean ± SD)	
			
					Cont.	Interv.	Cont.	Interv.	Cont.	Interv.	Cont.	Interv.
Ockenga et al. (59)	28/Germany	0.3	APACHE CT severity index	<72	10–18	6–16	5/14	4/14	1/14	0/14	25 (19–40)	21 (14–32)

Fuentes-Orozco et al. (60)	44/Mexico	0.4	APACHE CT severity index	24–48	17.5 ± 7.9	19.31 ± 12.62	16/22	9/22	5/22	2/22	26.59 ± 13.3	30.18 ± 10.42

Huang et al. (61)	32/China	0.099	APACHE	<72	–	–	2/18	2/14	0/18	0/14	20 ± 5	22 ± 5

Hajdu et al. (62)	45/Hungarian	0.5	–	48	–	–	–	–	3/21	0/24	15.9	10.6

Xue et al. (63)	76/China	20 g/day/person	APACHE CT severity index	<24	–	–	10/38	3/38	8/38	2/38	45.2 ± 27.1	28.8 ± 9.4

Singh et al. (67)	80/India	20 g/day/person	APACHE CT severity index	<120	7	7	19/39	21/41	6/39	5/41	11 (2–36)	12 (1–101)

*AD, the interval between admittance to ICU and start of enteral or parenteral nutrition; AP, acute pancreatitis; APACHE, acute physiology and chronic health evaluation; Cont., control; DOS, duration of hospital stay; EN, enteral nutrition; Interv., intervention; PN, parenteral nutrition*.

### Omega-3 Fatty Acids

Dietary polyunsaturated fatty acids have known immunomodulatory and other beneficial health-promoting effects. A prospective cohort study on the association of fish consumption and non-gallstone-related AP has suggested that total fish (fatty fish and lean fish combined) consumption may be associated with decreased risk of non-gallstone-related AP (68). A randomized prospective clinical trial assessing enteral formula enriched with ω-3 FAs in the treatment of AP suggested that EN supplemented with ω-3 FAs seemed to have clinical benefits based upon the shortened time of jejunal feeding and hospital stay (69). Subsequently, independent studies evaluated the effects of PN with ω-3 FA supplementation on severe AP. Wang et al. compared in a randomized, double-blind trial a total of 40 severe AP patients receiving PN with the same basal nutrients but different lipid compositions: soybean oil-/fish oil-based fat solutions. The study showed that patients with ω-3 FAs-supplemented PN had increased eicosapentaenoic acid concentrations and decreased pro-inflammatory cytokines, together with improved respiratory function and shortened continuous renal replacement therapy time, suggesting attenuated systemic responses to pancreatic and organ injury (70). A parallel study by the same group enrolling 56 patients who received isocaloric and isonitrogenous PN with fats of all ω-6 FAs or 4:1 ω-6:ω-3 FAs demonstrated that ω-3 FAs-supplemented PN elevated the IL-10 level and human leukocyte antigen-DR expression in severe AP patients (71). In accordance, during the initial stage of severe AP, parenteral supplementation with ω-3 fish oil emulsion was found to suppress SIRS, modulate the balance of pro-/anti-inflammatory cytokines and thus improve AP-associated severe conditions (72). Clinical studies on ω-3 FA supplementation have been summarized in Table 4. Although polyunsaturated FAs remain potential beneficial supplements with EN/PN, further larger trials are needed for formulations and confirmatory beneficial clinical effects.

**Table 4:** Characteristics of clinical trials on ω-3 FAs as the nutritional supplements in AP.

Reference	Subjects/regions	Dosage (g/kg BW/day)	Method of assessment	AD-EN or PN interval (h)	Duration of EN or PN (days)		Infectious complication (*n*/*N*)		Mortality (*n*/*N*)		DOS (days mean ± SD)	
			
					Cont.	Interv.	Cont.	Interv.	Cont.	Interv.	Cont.	Interv.
Lasztity et al. (69)	28/Hungary	3.3 g/day	APACHE CT severity index	<24	17.57 ± 10.52	10.57 ± 6.70	–	–	1/14	2/14	19.28 ± 7.18	13.07 ± 6.70

Wang et al. (70)	40/China	0.2	APACHE	<72	5	5	5/20	3/20	2/20	0/20	70.5 ± 9.1	65.2 ± 7.3

Wang et al. (71)	28/China	0.2	APACHE CT severity index	<72	5	5	9/28	6/28	2/28	0/28	–	–

*AD, the interval between admittance to ICU and start of enteral or parenteral nutrition; AP, acute pancreatitis; APACHE, acute physiology and chronic health evaluation; Cont., control; DOS, duration of hospital stay; EN, enteral nutrition; Interv., intervention; PN, parenteral nutrition; ω-3 FAs, omega-3 fatty acids*.

### Vitamins

Oxidative stress is involved in the onset of AP and also in the development of the systemic inflammatory responses, being glutathione depletion, xanthine oxidase activation, and thiol oxidation in proteins critical features of the disease in the pancreas. Vitamins as important immunonutrients and antioxidants have been inversely associated with AP (73). Plasma concentrations of vitamin A and vitamin C were found significantly lower in AP patients than in healthy controls (*P* < 0.05) (74). Recently, vitamin D, mainly from the milk products, has been inversely associated with gallstone-related AP (73). Vitamin supplementation assessed in combination with other antioxidants or in vitamin-only therapy has been evaluated earlier and yielded mixed outcomes. A multicenter randomized, double-blind, placebo clinical trial by Siriwardena et al. concluded that use of intravenous combination antioxidant therapy containing vitamin C (*N*-acetylcysteine, selenium, vitamin C) was not justified to continue in clinical severe AP (75). Subsequently, another group comparing vitamin C, *N*-acetylcysteine, antoxyl forte antioxidant combination with standard medical treatment in early AP patients suggested that antioxidant supplementation could decrease the length of hospital stay and complications in patients with early AP, but this hypothesis needed to be supported by a larger clinical trial (76). With respect of vitamin-only antioxidant therapies, a study involving 84 AP patients and 40 healthy subjects in China on high-dose vitamin C has demonstrated that it has therapeutic efficacy on the disease and proposed the potential mechanisms to be promoting anti-oxidizing capability in patients, blocking lipid peroxidation and improving cellular immune function (77). In contrast, multiple vitamins-based antioxidant therapy (vitamin A, vitamin C, and vitamin E) in a single-center randomized study involving 39 patients has not been proven beneficial in patients with established severe AP (78). Collectively, data so far on vitamin therapy in AP (Table 5) have been mixed and should be carefully evaluated for dosing and timing of intervention for potential promising outcomes in clinical use.

**Table 5:** Characteristics of clinical trials on vitamins as the nutritional supplements in AP.

Reference	Subjects/region	Vitamin(s) tested	Dosage (g/kg BW/day)	Method of assessment	Duration of EN or PN (days)		Mortality (*n*/*N*)		DOS (days mean ± SD)	
		
					Cont.	Interv.	Cont.	Interv.	Cont.	Interv.
Siriwardena et al. (75)	43/UK	Vitamin C + *N*-acetylcysteine, selenium	For vitamin C, 2 g/day for 2 days, 1 g/day (continued for up to day 7)	APACHE	7	7	0/21	4/22	14.3 (15.7)	20.4 (24.4)

Sateesh et al.(76)	53/India	Vitamin C, *N*-acetyl cysteine, and antoxyl forte	Vitamin C 500 mg, *N*-acetyl cysteine 200 mg 8 hourly and antoxyl forte 1 capsule hourly	APACHE CT severity index	–	–	0/30	1/23	10.3 ± 7	7.2 ± 5

Du et al. (77)	84/China	Vitamin C	10 or 1 g/day (con)	Detection of clinical, biochemical, and immunological markers	5	5	–	–	13.45 ± 3.21	9.34 ± 4.24

Bansal et al. (78)	39/India	Vitamin A, vitamin E, vitamin C	Vitamin C (1,000 mg in 100 ml saline), vitamin E (200 mg oral), and vitamin A (10,000 IU)	APACHE CT severity index	14	14	2/20	0/19	15.1 ± 5.43	12.8 ± 3.9

*AP, acute pancreatitis; APACHE, acute physiology and chronic health evaluation; Cont., control; DOS, duration of hospital stay; EN, enteral nutrition; Interv., intervention; PN, parenteral nutrition*.

## Conclusion and Future Perspectives

In most patients, an oral soft or solid diet can be beneficial if tolerated. When oral feeding is not tolerated for a few days, enteral feeding through a nasogastric or nasojejunal feeding tube should be attempted within the first 72 h of administration. PN should be minimized for its risks of infection and other complications. Only if enteral route is not available or tolerated, PN may be considered. Overall, nutritional support plays a critical role in clinical management of severe AP, although the optimal timing remains unclear. Predicting the nutritional tolerance of patients with AP remains challenging as the current evaluation system needs to be improved. Various nutritional supplement(s) together with PN or EN with currently mixed clinical outcomes is a subject of interest for future evaluation and may lead to promising outcomes. In addition, given its heterogeneous etiological factors and varying clinical manifestations, precision medicine, although not much applied in the condition, remains as a temping approach to optimize clinical outcomes on classified individuals based on susceptibility to the condition and its systemic complications.

## Author Contributions

JS designed the subject content of the review article. L-LP, JL, MS, and JS conducted initial search of literature, drafted the manuscript, and prepared the figures and tables. MB gave the constructive comments and critically reviewed the manuscript. JS had primary responsibility for final content. All authors read and approved the final manuscript.

## Conflict of Interest Statement

The authors declare that the research was conducted in the absence of any commercial or financial relationships that could be construed as a potential conflict of interest.

## Abbreviations

AP, acute pancreatitis; ERCP, endoscopic retrograde cholangiopancreatography; SIRS, systemic inflammatory response syndrome; MODS, multiple organ dysfunction syndrome; ω-3 FAs, omega-3 fatty acids; PN, parenteral nutrition; EN, enteral nutrition; TPN, total parenteral nutrition.
