# Corrigendum: Recent Advances on Nutrition in Treatment of Acute Pancreatitis

**DOI:** 10.3389/fimmu.2018.00849

**Published:** 2018-04-23

**Authors:** Li-Long Pan, Jiahong Li, Muhammad Shamoon, Madhav Bhatia, Jia Sun

**Affiliations:** ^1^School of Medicine, Jiangnan University, Wuxi, China; ^2^State Key Laboratory of Food Science and Technology, Jiangnan University, Wuxi, China; ^3^Nutrition and Immunology Laboratory, School of Food Science and Technology, Jiangnan University, Wuxi, China; ^4^Inflammation Research Group, Department of Pathology, University of Otago, Christchurch, New Zealand

**Keywords:** clinical management of acute pancreatitis, nutritional interventions, probiotics, prebiotics, vitamins, amino acids, omega-3 fatty acids

In the published article, there was an error in affiliation [1,2]. Instead of “^1^Department of Pharmacology, School of Pharmacy, Fudan University, Shanghai, China, ^2^School of Medicine, Jiangnan University, Wuxi, China”, it should be “^1^School of Medicine, Jiangnan University, Wuxi, China”. The authors apologize for this error and state that this does not change the scientific conclusions of the article in any way.

The original article has been updated.

## Conflict of Interest Statement

The authors declare that the research was conducted in the absence of any commercial or financial relationships that could be construed as a potential conflict of interest.

